# Increased knowledge makes a difference! – general practitioners’ experiences of pictorial information about subclinical atherosclerosis for primary prevention: an interview study from the VIPVIZA trial

**DOI:** 10.1080/02813432.2021.1882083

**Published:** 2021-02-11

**Authors:** Anna Bengtsson, Kristina Lindvall, Margareta Norberg, Eva Fhärm

**Affiliations:** a Department of Epidemiology and Global Health, Umeå University, Umeå, Sweden; b Department of Public Health and Clinical Medicine, Umeå University, Umeå, Sweden

**Keywords:** Cardiovascular disease, consultation process, family practice, pictorial information, qualitative research, risk

## Abstract

**Objectives:**

To explore how pictorial information on subclinical atherosclerosis affects GPs’ perception of patient cardiovascular disease (CVD) risk, their communication with patients, and GPs’ attitude to the treatment of CVD risk factors.

**Design, setting and subjects:**

Fifteen individual interviews were conducted between March 2014 and December 2016, with GPs who had received pictorial information regarding their patients’ subclinical atherosclerosis. The pictorial information was also received by the patients together with written information regarding atherosclerosis and CVD risk prior to the appointment with their GP. The interviews were recorded, transcribed and analyzed using qualitative content analysis.

**Results:**

Three categories were identified in the analysis. *Increased knowledge makes a difference:* When patients had more in-depth knowledge regarding atherosclerosis, the consultation became more patient-centered and moved towards shared decision making. *This is real, not just a number:* GPs described their risk assessment and the patient’s risk perception as more accurate with pictorial information about subclinical atherosclerosis. *How to deal with the result – A passive to active approach:* Some GPs acted promptly on the pictorial information while others took no action.

**Conclusion and implications:**

Pictorial information regarding patients’ subclinical atherosclerosis affected GPs’ assessment of CVD risk. The communication shifted towards shared decision-making although the GPs’ attitude to the result and treatment of CVD risk factors varied. Informing patients about examination results, both in writing and pictures, prior to a consultation can facilitate shared decision making and enhance preventive measures.

**Trial registration:**

https://clinicaltrials.gov/ct2/show/NCT01849575.

**KEY POINTS**
Providing pictorial information about carotid ultrasound results and information regarding atherosclerosis to GPs and patients affects primary prevention:
•Informing patients about examination results prior to a consultation can be useful in clinical practice to enhance preventive measures
•GPs experienced that increased patient knowledge resulted in a more patient-centered consultation and improved shared decision-making
•GPs described their risk assessment and patients’ risk perception as more accurate with pictorial information about subclinical atherosclerosis

## Introduction

Cardiovascular disease (CVD) is the leading cause of death worldwide [1]. CVD is mainly an atherosclerotic disease and up to 95% of occurrences are caused by modifiable factors [2,3]. Increased carotid artery intima-media thickness (CIMT) and presence of plaque are early signs of atherosclerosis and associated with future CVD [4,5]. Prevention can reduce risk, but for any intervention to be effective the communication of the risk must be correctly perceived and have sufficient impact on recommended preventive measures.

In GP consultations, graphical presentation of data could facilitate and save time in communicating risk [6]. Physicians appreciate colors to emphasize severity and simple comparative information, such as a thermometer scale, to motivate patients to modify behavior [7]. If practitioners can make patients adequately aware of their risk, this can encourage them to perform preventive measures to reduce that risk, especially if the risk is high [8].

An additional strategy to conventional risk factor-based assessment would be to assess and communicate CVD risk in pictorial form based on the prevalence and extent of the patients’ subclinical atherosclerosis. Subclinical atherosclerosis can be detected by ultrasound of the carotid arteries. New automated ultrasound systems have been developed, making ultrasound examinations with high reproducibility feasible and cost-effective within community medicine [9].

This study is part of the VIPVIZA trial (**VI**suali**Z**ation of asymptomatic **A**therosclerotic disease for optimum cardiovascular prevention – a randomized controlled trial nested in the **V**ästerbotten **I**ntervention **P**rogram). Recently published 1-year follow-up data provides evidence of the contributory role of pictorial presentation of subclinical atherosclerosis to reduce CVD risk [10].

We aimed to explore how pictorial information of patients’ subclinical atherosclerosis provided to patients and physicians, affects GPs’ perception of patients’ risk, their communication with patients and their attitudes to and treatment of CVD risk factors.

## Material and methods

This was a qualitative study using individual semi-structured interviews with fifteen GPs practicing in Västerbotten County, Sweden. The interviews were conducted between March 2014 and December 2016. The GPs’ patients had received conventional CVD risk factor assessment and information through the Västerbotten Intervention Programme (VIP) [11]. Both primary and secondary CVD prevention are common concerns in Swedish primary health care.

In VIP, all Västerbotten residents are invited to their local primary care center during the year in which they turn 40, 50 or 60 years, in order to undergo CVD risk factor screening, together with individual counseling and a health promotion discussion with trained nurses[11]. On this visit, prospective VIPVIZA participants are informed of the trial and invited to participate. Inclusion criteria for VIPVIZA were (1) age 40 and family history of CVD before age 60 among first-degree relatives; (2) age 50 and at least one classical CVD risk factor or (3) age 60. Participants in VIPVIZA were randomized into two equal groups: intervention and control.

All participants underwent a carotid ultrasound examination with portable ultrasound equipment to detect plaques and measure cIMT. The method has previously been described in detail [10]. Participants with significant carotid stenosis (>50%) were excluded from VIPVIZA and referred for special care (*n* = 22, as compared to included *n* = 3532). In the case of technical problems, difficulties in evaluating the images or confirming suspected carotid stenosis, the patient may also undergo an extended ultrasound examination; this was required for only a minority of patients. All ultrasound examinations followed a strict protocol with automated IMT measurements at predefined angles. The presence of plaque was defined at the occasion of examination [10]. All participants, both control and intervention groups, will be re-examined by ultrasound after three years and all participants will receive follow-up pictorial information.

In the intervention group, participants and their GP were informed of the extent and severity of the participants’ atherosclerosis in pictorial form according to the VIPVIZA protocol [10]. No information about the ultrasound results was given to the control group and their respective GPs.

**Figure 1.**
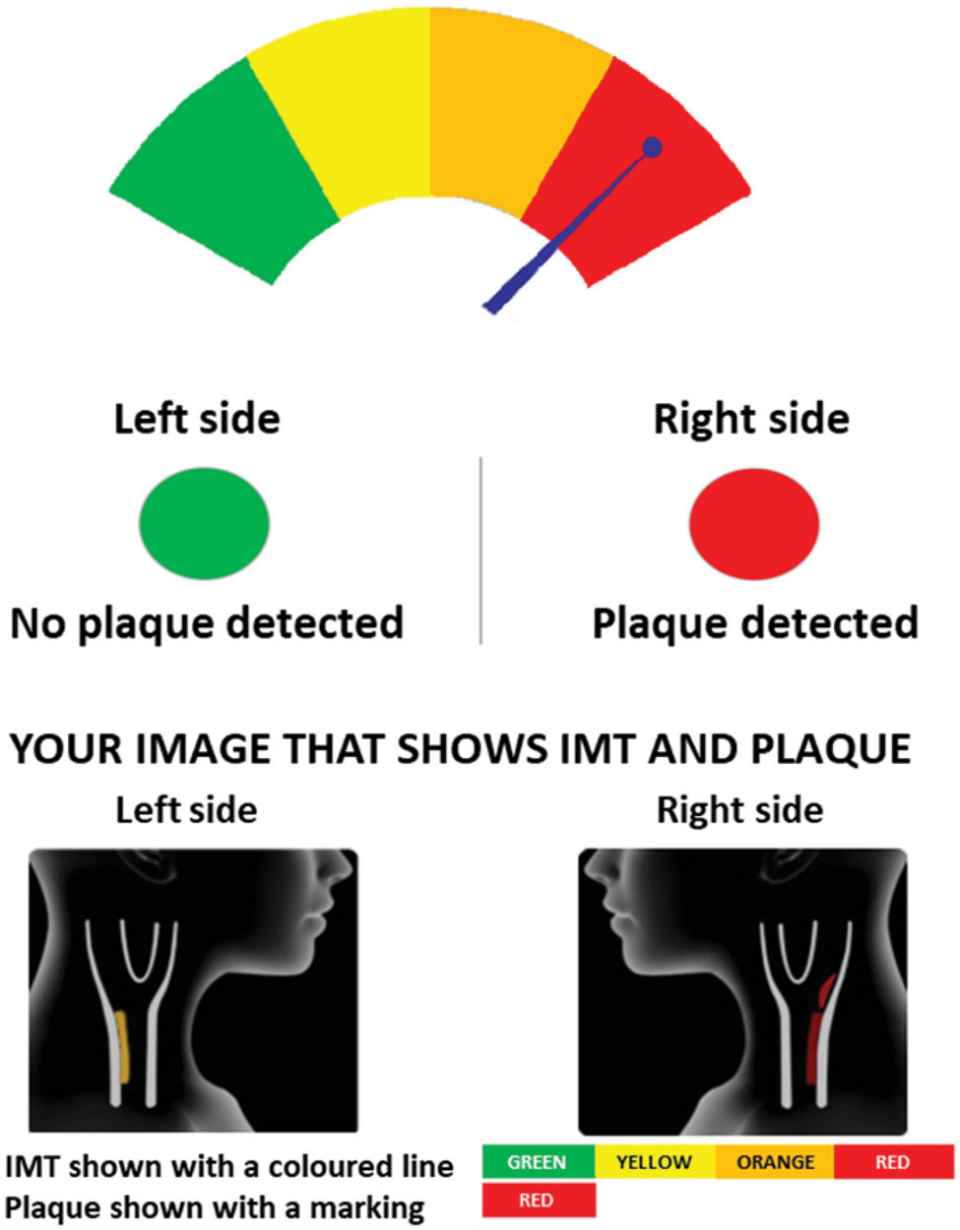
Pictorial information based on ultrasound examination of the carotid arteries presented to GPs and their patients.

The information provided to the intervention group and their GPs included a stylized picture of the individual’s carotid arteries. For each side, the presence of plaque was presented as a red circle, while a green circle indicated that plaque was not observed. A gauge presented the CIMT compared to a reference population, running from green (comparable with individuals aged ≤10 years younger than the participant) to red (comparable with individuals aged ≥10 years older than the participant) [12] (Figure 1). In addition, written information explaining the modifiable nature of atherosclerosis and how to minimize atherosclerosis development through a healthy lifestyle and adherence to preventive medications was included. After 2–4 weeks, patients received an additional follow-up phone call with a nurse trained in motivational interviewing techniques to ensure the results were correctly understood, and, if necessary, to give additional information and reduce anxiety.

Moreover, GPs received a leaflet with a brief description of the VIPVIZA study and instructions to interpret plaques as ‘very high risk’ according to the SCORE system, the European Society of Cardiology (ESC) Systematic Coronary Risk Evaluation assessment model. The web address to the ESC guidelines for primary prevention of CVD [13,14] was also given, together with information that some GPs would be contacted for a qualitative interview study.

A letter asking GPs to participate in this study was sent to health centers. Further recruitment was conducted via phone calls to GPs, consulting nurses or health care center superiors. To be included, the GP needed to have received ultrasound results for at least three different patients. The characteristics of participating GPs are shown in Table 1.

**Table 1.** Characteristics of the participating GPs who were interviewed about their experience of VIPVIZA.

Sex	
Female	4
Male	11
Patient recruitment area:	
Urban	9
Rural	6
Experience as a GP	
<5 years	6
>5 years	9

The first author (AB) conducted all interviews. AB had no relationship to any of the GPs. She introduced herself as a PhD student and GP in advance of the interview. Before the interviews began, the GPs were informed about their right to withdraw from the study at any time. This information was followed by general questions to ascertain the number of years practicing as a GP, whether the practice was urban or rural and the number of VIPVIZA results received. The GPs received no financial compensation for participation.

The interviews lasted 25–45 min and were conducted at a location selected by the GP. A semi-structured interview guide (Table 2) was developed based on the VIPVIZA trial’s conceptual framework presented in the Study protocol and available at https://clinicaltrials.gov/ct2/show/NCT01849575, literature review of the field and the author’s clinical experience being a GP. The first question asked in all interviews was ‘What is your experience of the VIPVIZA trial?’ After the first three interviews, the guide was revised based on the initial analysis with one questions added (‘What do you remember from the VIPVIZA result?’) and one question removed (‘What is cardiovascular risk for you?’, together with probing questions covering experiences from cardiovascular risk assessment without VIPVIZA results). The interviews were recorded and transcribed verbatim and analyzed using qualitative content analysis [15].

**Table 2.** Interview guide followed during interviews with GPs to explore their experiences of participation in VIPVIZA.

Main topics	Probing questions
General experience of VIPVIZA	What is your general impression of VIPVIZA?
	What do you remember of the results from VIPVIZA?
	Can you see any advantages or disadvantages with VIPVIZA?
Experience of the VIPVIZA result with CIMT and plaque	Does this information add anything to your practice? Why or why not?
	Does the result affect how you treat the patient? How?
	Does your perception of the patients’ CVD risk change with the VIPVIZA result?
	If you could choose, would you like to have the result or not?
Communication of risk	What is your experience of the patient having the same information as you have regarding the ultrasound examination?
	Does the result affect how you communicate CVD risk? If yes, in what way?

The authors represented a multidisciplinary research team from the fields of general practice, public health and nutrition with 2–35 years of experience in clinical work from different regions in Sweden. The first author (AB) is a PhD-student, KL PhD, MN associate professor and EF PhD. When the interviews were completed, each author read the text of the first four interviews and identified ‘units of meaning’ which were then condensed. The process of analysis included both naïve reading of the transcribed interviews to obtain a sense of the whole and interpretation of the latent content of the interviews. An illustration of the analytical process is given in Table 3. The first author then coded the remaining interviews. In a series of meetings between the authors, the findings were interpreted and the condensed ‘units of meaning’ were abstracted and labelled with codes. Categories were identified and refined, and the codes were sorted into categories. No new categories were revealed and saturation appeared to be achieved after analysis of eleven interviews. The four remaining interviews were listened through twice, but no new information emerged. All authors were involved in all steps of the analytical process, AB wrote the manuscript in close collaboration with the other authors.

**Table 3.** Illustrative description of the analytical process to extract categories from raw interviews exploring GPs experience of VIPVIZA.

Meaning unit	Condensed meaning unit	Code	Categories
The communication is affected because the patient is more conscious	The communication is affected when patient is more conscious	Increased patient knowledge effects communication	Increased knowledge makes a difference
I think patients becomes more involved in their treatment. More aware of their disease and more aware of their treatment and compliance. That is what makes patient more motivated to follow up their medication and also know how serious the disease is. It is not just like that.	Patients becomes more involved in their treatment. More treatment and compliance awareness when knowing the seriousness of the disease. Increases motivation to follow up.	Increased knowledge of disease seriousness gives more motivation and compliance awareness	
It is after all an examination with a clear result, so it is clear to the patient anyway	Examination with clear results to patients anyway	Clear result to patient	This is real, not just a number
Oh my god, your blood pressure is 108 systolic and total cholesterol on 3,5 and still you have this	Low blood pressure and cholesterol and still subclinical atherosclerosis	Low risk factor burden but still subclinical atherosclerosis	
I read through it and then look at the gauge and then look at the plaque like that but then I put it for scanning. I don’t do anything more with it	Reads text, looks at the gauge and presence of plaque. Send for scanning. No action	Reads the results with no action taken	How to deal with the result- from passive to active approach
This is really good, one finds patients at risk and one can do something about it and give them treatment	Positive, finds patients at risk, do something about the risk with treatment	Acts on the result with treatment	

*Note*. The table displays examples of meaning units, condensed meaning units, codes generating the final three categories.

## Results

In the analysis of the interviews with GPs, three categories were identified; ‘Increased knowledge makes a difference’, ‘This is real, not just a number’ and ‘How to deal with the result – From passive to active approach’.

Each category is described next.

### Increased knowledge makes a difference

The patients received information and results from VIPVIZA before seeing their GPs. This was in general perceived as an advantage as the discussion was described as more profound since the patient had a more advanced knowledge regarding their CVD risk and atherosclerosis in general. With increased patient knowledge, the GPs found their role more consultative than instructive. The encounter became an issue of confirming and giving feedback, collaboration rather than just the GPs informing the patient of what to do.

I ask, what information have you got from the study? Then I do not have to fantasize what to say, I get exact information from the patient. Then I give feedback to the patient. It is easier to give feedback than starting from scratch. It is something different compared to when beginning with patients that you have to motivate to change (GP5)

They are more interested in knowing, many patients are up-to-date and are well-informed, they know what it is all about. They are positive in their attitude towards treatment, obviously, because they realize they are at risk (GP9)

Some GPs emphasized that the patient should participate in their treatment and shared decision-making was mentioned as preferable. When the patient participated in VIPVIZA, GPs found that the attention was transferred from the doctor to the patient.

So the consultation becomes reasoning between me and the patient: this is the information we have, these are the recommendations I give you on the basis of that information (GP2)

When the patient had more knowledge, less time was spent on motivation. The GPs described most VIPVIZA patients as motivated to undertake preventive measures, although there were patients who did not make changes. This situation was familiar to the GPs and could sometimes cause frustration.

Well it differs how they accept the result, some just put their head in the sand while others act by making changes (GP5)

It is not up to me if they want to continue to live GP 9 continues I give them the tools, medications, information and so on. But for some, it goes in in one ear and out through the other. They don’t care and those are the most difficult ones (GP 9)

### This is real, not just a number

The GPs’ perception of the patients’ CVD risk was altered by the pictorial representation of the patients’ results, which was considered valid and the consequences of refraining from preventive actions were clear.

It is black and white, it is not just a high value (GP1)

CVD prevention was considered important but difficult. GPs using the VIPVIZA result when communicating risk found it useful when they tried to reach out to and motivate their patients. The patients understood normal atherosclerotic disease progression and their own personal CVD risk more accurately than with normal care.

Many patients accept this information in a way that makes you feel that you have reached them, they understand what it is all about (GP9)

The GPs advocated lifestyle changes and pharmaceutical treatment of hypercholesterolemia and hypertension to prevent CVD. However, explaining the association between risk factors and atherosclerosis to patients was considered difficult. Some GPs considered the carotid artery images helpful when explaining the association between risk factors and atherosclerotic disease.

I believe they think it is only high blood pressure and high cholesterol, that this does not mean much (GP1)

I have more facts regarding their health, and if there is something atypical then I have this as support. I find it useful (GP9)

In general, GPs regarded results showing the presence of plaque or a red indicator for CIMT to be more serious than their patients considered them. The GPs’ explanation for this was that doctors were more likely to understand the clinical implications. The results were sometimes a surprise to the GPs and led to questions about why some individuals had more atherosclerosis than expected based on risk factors. The GPs’ perception of the patient’s risk often changed based on the result.

Often it comes as a surprise to them and to me but I don’t think they understand and interpret it the same way…. They don’t see it as seriously as I might do (GP9)

Some GPs found that the straightforward information could cause stress and anxiety to patients. However, some degree of stress or anxiety was not necessarily considered to be a bad thing by the doctors, as the patients’ motivation to behavioral change increased when the severity of atherosclerosis became clear.

I experience an increased awareness regarding their treatment but there is also a small amount of patient anxiety, for better or worse. Increased anxiety can be good because you get more motivated to carry out your treatment (GP5)

Some doctors met no reactions from patients while others encountered powerful reactions with fear and anxiety. After explaining the results and emphasizing that atherosclerosis is a modifiable process, the patient was empowered to make changes.

One woman said, – My life is destroyed, I have found out that I am 10 years older than I thought I was. It was a shock to her… but then the GP continued Well, at the end the patient still thought it was necessary. Painful but necessary (GP3)

### How to deal with the result – from passive to active approach

Some GPs were more prone to treat their patients pharmacologically when there were signs of atherosclerosis, particularly presence of plaques. Others hesitated, concerned that treating the patient would cause over-prescription.

When I do traditional risk evaluation then I only have the patients’ profile and values, I am more used to that. Now I am in a gray-zone, when you have more information than before and wants to act on it. But then you don’t know if you do the correct thing or am I over-prescribing? (GP 6)

The information made some GPs more proactive and was sometimes used as an instrument to motivate the patient to make lifestyle changes and to evaluate the need for medication.

I have a more solid base on which to tell them and motivate them that their blood pressure needs to be regulated, therefore you need more medication. Physical activity and lipids are also important (GP5)

When the patient’s results were entirely green (i.e. low risk for CVD), then on-going preventive medication, if any, was not discontinued. The GPs argued that if the reason for treatment was once present and if there had been no side effects, a green result was considered to be, at least in part, the fruits of successful medication.

You don’t know if they are green because of successful treatment. If the patient feels good under treatment one should only say, or think, that this looks great (GP5)

In general, the workload was heavy and a few GPs described being too short of time to look into the VIPVIZA results and act on them. Some healthcare centers had developed their own guidelines on how to deal with the VIPVIZA results, with nurses handling low/normal risk results, while high-risk results were handled by physicians. Others had made a collegiate decision not to change the pharmacological treatment based on the VIPVIZA result, arguing that VIPVIZA is research and results on hard endpoints were still not available. In general, GPs interviewed in the first year of VIPVIZA seemed to be more hesitant about the results and their role compared to the GPs interviewed during the third year.

## Discussion

The main findings from this study were that a difference was made when both patients and GPs received a pictorial representation of the patient’s subclinical atherosclerosis together with written information about the atherosclerotic process. When patients received the ultrasound result before the consultation, the consultation moved towards patient-centeredness and shared decision-making. The pictorial information about subclinical atherosclerosis had an impact on how GPs interpreted the CVD risk. GPs described their risk assessment, as well as patients’ risk perception, as more accurate with pictorial information; the information was concrete, not just a number. Nevertheless, the GPs had different attitudes towards the treatment of risk factors based on the VIPVIZA results, from a passive to active approach.

Our results suggest that receiving a result based on actual atherosclerosis as a complement to conventional risk factor assessment and information could be more informative and can enhance CVD preventive actions. This finding is in line with another study using coronary calcium scoring to demonstrate subclinical atherosclerosis [16]. When patients with a high risk of CVD were interviewed about their experience of lifestyle change after participating in a lifestyle program, increased knowledge was an important factor for change [17].

It was in general considered positive that GPs and patients both received the pictorial information prior to the consultation. To receive a result based on actual atherosclerosis as a complement to conventional risk factor assessment and information could be more informative and can enhance CVD preventive actions. A Cochrane review regarding decision aids for people facing health treatment or screening decisions, found in four out of five studies that participants discussed the choice with the physician to a greater extent when exposed to the decision aid prior to the consultation [18]. This supports our result; presenting the findings from an examination, together with information regarding the condition and treatment options, favors a dialogue between the patient and the physician.

Several GPs stated that shared information had a beneficial impact on the communication and led to a more shared decision-making process. The information exchange became a two-way process [19], instead of the GP informing the patient. VIPVIZA changed the focus from the doctor to the patient [20]. Similar findings were observed in primary care consultations when patients had used a self-management support system to report blood pressure measurements for hypertension management. With the self-management support system, the patients’ understanding of hypertension increased and the patients’ contribution during the consultation grew, generating an increased partnership and a more person-centered consultation [21]. A majority of the GPs found that the patients had more knowledge than ordinary patients regarding the atherosclerotic process and the potential effect of preventive measures. The dialogue became an issue of confirming the patients’ understanding and a deal regarding treatment was arrived at more easily. GPs also described patients as more motivated to adopt the treatment modalities. In chronic diseases such as atherosclerosis, preventive actions and treatments are mostly based on patients’ personal responsibility, requiring an active role from the patient [22]. Many GPs found an enhanced collaboration with patients in VIPVIZA regarding active prevention. Nevertheless, some GPs had encountered situations when the patient did not have the same CVD risk perception as the doctor; in this instance, the VIPVIZA result was then used to explain CVD risk and motivate the patient to undertake preventive actions. Some GPs had observed patients who reacted with anxiety and/or fear after receiving the results. The impression was that some degree of fear seemed to motivate patients to take action. Fear has been described as motivating, as long as the individuals believe they have the ability to protect themselves, otherwise it may lead to anxiety and defensive avoidance [23].

The pictorial result was in general considered clear and reliable. The graphic information regarding plaque and the color gauge relating CIMT to vascular age was recalled by most GPs as being of substantial benefit, ‘not just a number’. Other studies support the use of graphs and visual aids as a complement to numerical and verbal communication of risk [24] to optimize understanding and also improve the physician-patient relationship [25].

Many GPs changed their practice in relation to the VIPVIZA report, while others were less pro-active. This is in line with the theoretical framework ‘Diffusion of Innovations’, which comprises those referred to as innovators, early adopters, late adopters and laggards. This theory concerns the conditions and processes by which people in a social system adopt an innovation [26–29]. In general, GPs interviewed in the first year of VIPVIZA seemed to be more hesitant about the results and their role, compared to the GPs interviewed during the third year. This could reflect the fact that GPs interviewed during the third year had had time to adapt to the VIPVIZA results. Whether GPs change their practice over time will be further evaluated in quantitative longitudinal analyses within VIPVIZA.

In conclusion, pictorial information regarding patients’ subclinical atherosclerosis affected GPs’ assessments of CVD risk. The communication shifted towards shared decision-making, although the GPs’ attitudes to the result and treatment of CVD risk factors varied. We found that informing patients about examination results, both in writing and pictures, prior to a consultation can facilitate shared decision making and enhance preventive measures.

## Strengths and limitations

GPs participating in this study were heterogeneous in terms of work experience, gender, practice location (urban or rural) and timing of the interview during the period of entry into the trial, which strengthened the credibility of the study [15]. However, a great effort was needed to enroll GP participants. Therefore, GPs agreeing to participate might not be representative of all GPs, but nevertheless, broad variations in experiences and attitudes were identified and the notion of saturation was fulfilled.

Saturation was determined by an abductive process where data collection and analysis were conducted alternately [30]. The methodology used (qualitative content analysis) is well described and established. Transferability was strengthened through different academic backgrounds and experiences by the authors. The study was performed over 2.5 years which would make the study more consistent over time and improve its dependability [15].

## Meaning of the study

To provide pictorial information of subclinical atherosclerosis to patients and their GPs has the potential to improve the primary prevention of CVD and enhanced shared decision-making. The concept of sending test results and information regarding their condition to patients prior to the consultation is a new approach, to our knowledge rarely studied before. This approach could be applicable in several different clinical situations, for example, diabetes.
